# Nomogram for pneumonia prediction among children and young people with cerebral palsy: A population-based cohort study

**DOI:** 10.1371/journal.pone.0235069

**Published:** 2020-07-06

**Authors:** Tsu Jen Kuo, Chiao-Lin Hsu, Pei-Hsun Liao, Shih-Ju Huang, Yao-Min Hung, Chun-Hao Yin

**Affiliations:** 1 Department of Stomatology, Kaohsiung Veterans General Hospital, Kaohsiung, Taiwan; 2 Department of Marine Biotechnology and Resources, National Sun Yat-sen University, Kaohsiung, Taiwan; 3 School of Dentistry, Chung Shan Medical University, Taichung, Taiwan; 4 Center of Health Management, Kaohsiung Veterans General Hospital, Kaohsiung, Taiwan; 5 Center for Geriatrics and Gerontology, Kaohsiung Veterans General Hospital, Taiwan; 6 Department of Family Medicine, Kaohsiung Veterans General Hospital, Kaohsiung, Taiwan; 7 Department of Otolaryngology, Head and Neck Surgery, Kaohsiung Veterans General Hospital; 8 School of Medicine, National Defense Medical Center, Taipei, Taiwan; 9 Department of Pediatrics, Kaohsiung Veterans General Hospital, Kaohsiung, Taiwan; 10 Department of Internal Medicine, Kaohsiung Municipal United Hospital, Kaohsiung, Taiwan; 11 School of Medicine, National Yang Ming University, Taipei, Taiwan; 12 Yuhing Junior College of Health Care and Management, Kaohsiung, Taiwan; 13 Department of Emergency Medicine, Kaohsiung Veterans General Hospital, Kaohsiung, Taiwan; 14 Department of Medical Education and Research, Kaohsiung Veterans General Hospital, Kaohsiung, Taiwan; Universitat de les Illes Balears, SPAIN

## Abstract

**Background:**

Pneumonia is the leading cause of death among children and young people (CYP) with severe cerebral palsy (CP). Only a few studies used nomogram for assessing risk factors and the probability of pneumonia. Therefore, we aimed to identify risk factors and devise a nomogram for identifying the probability of severe pneumonia in CYP with severe CP.

**Methods:**

This retrospective nationwide population-based cohort study examined CYP with newly diagnosed severe CP before 18 years old between January 1^st^, 1997 and December 31^st^, 2013 and followed them up through December 31^st^, 2013. The primary endpoint was defined as the occurrence of severe pneumonia with ≥ 5 days of hospitalization. Logistic regression analysis was used for determining demographic factors and comorbidities associated with severe pneumonia. These factors were assigned integer points to create a scoring system to identify children at high risk for severe pneumonia.

**Results:**

Among 6,356 CYP with newly diagnosed severe CP, 2,135 (33.59%) had severe pneumonia. Multivariable logistic regression analysis revealed that seven independent predictive factors, namely age <3 years, male sex, and comorbidities of pressure ulcer, gastroesophageal reflux, asthma, seizures, and perinatal complications. A nomogram was devised by employing these seven significant predictive factors. The prediction model presented favorable discrimination performance.

**Conclusions:**

The nomogram revealed that age, male sex, history of pressure ulcer, gastroesophageal reflux, asthma, seizures, and perinatal complications were potential risk factors for severe pneumonia among CYP with severe CP.

## Introduction

Cerebral palsy (CP), a crucial global public health concern, is the most common physical disability in early childhood [1–3]. The worldwide prevalence and incidence of CP are approximately 2–2.5 cases per 1,000 live births [2], and approximately 1–4 cases per 1,000 live births, respectively.[4] CP is clinically characterized by nonprogressive motor and cognitive and perceptive impairments secondary to the injury of the immature brain, exerting considerable influence on health outcome, quality of life, and life expectancy [5–7].

Studies have revealed that children with CP frequently develop multisystemic disorders, including respiratory [8], digestive, musculoskeletal, neurologic, and nutritional diseases, which require hospitalization [9]. Children with CP are particularly vulnerable to respiratory infection complications and have a higher risk of mortality [10, 11]. The primary reason for hospitalizations [12] and the leading cause of death among younger individuals with CP is pneumonia [4]. More than half (53%–58.6%) of the deaths among children with CP were attributed to respiratory infection and failure [13, 14].

CYP with CP with gastroesophageal reflex disease (GERD), oromotor dysfunction, seizures, poor nutritional status, or kyphoscoliosis can easily cause respiratory infection and disease during hospitalization [4, 10, 15]. Other risk factors for pneumonia include younger age, underweight, and lower maternal educational status [16]. Although factors associated with pneumonia have been investigated among children with CP, limited studies have focused on the risk factors for severe pneumonia in Asian children with CP using nomogram predictive models.

Nomograms, raphical depictions of predictive statistical models, have been developed and validated to assess various diseases outcomes, mainly cancer outcomes [17, 18]. They have consistently presented more favorable performance characteristics than other available options [19]. Moreover, Kawasaki et al. predicted postoperative pneumonia after major abdominal surgery using a nomogram [20]. This clinical tool has also been validated in a research focused on children with severe pneumonia [21].

Hence, the present study aims to identify factors associated with pneumonia among CYP with severe CP, and to establish a predicting nomogram based on population-based administrative data in Taiwan.

## Methods

### Data source

Data from the Taiwan’s National Health Insurance Research database (NHIRD) were used, which were released by the National Research Institutes for research purposes. Taiwan’s National Health Insurance (NHI) was established in 1995, which covered 99.6% of the whole population up to 2011. The advantages of using the NHIRD for research purposes have been described in previous literature [22]. The NHIRD registry consists of data of patients’ demographic characteristics, all types of medical visits, the medical costs for reimbursement; codes of diseases diagnosed; laboratory tests and procedures performed; and prescriptions prescribed. This study was approved by the Institutional Review Board of Kaohsiung Veterans General Hospital, Kaohsiung, Taiwan (VGHKS15-CT12-01). Because all personal identifications are replaced with surrogate numbers, no informed consent was required from the study population. NHIRD has a registry for catastrophic illnesses patient database (RCIPD) [23], covering approximately 30 diseases including CP.

### Study cohort

This study is a retrospective cohort study analyzing newly diagnosed YCP with severe CP between January 1^**st**^,1997 to December 31^**st**^, 2013.

### Definition of severe CP

Severe CP diagnosis in this study were based on the International Classification of Disease, Ninth Revision, Clinical Modification (ICD-9-CM) diagnosis codes 343.X. and catastrophic illness certificate.

To obtain a catastrophic illness certificate in Taiwan, first, CP diagnosis must be confirmed by the pediatric neurologists or rehabilitation specialists. Their subspecialist licenses were certified by the Ministry of Health and Welfare. Furthermore, they were claimed to train for additional 2–3 years and pass specialty test to become subspecialists. Second, in addition to clinical diagnosis, neonatal magnetic resonance imaging was also used for detecting cerebral palsy when clinical uncertainty. Third, the certification must be verified by the National Health Insurance Administration. Lastly, only those who have proven definite CP diagnosis with moderate to severe physical or mental disability by the designated hospitals can apply for a catastrophic illness certificate.

The disability qualification defined by two or more significant functional impairments according to the International Classification of Functioning (ICF), Disability and Health as follows: 1. Cognition, coherence, and psychological level; 2. Joint mobility (i.e., upper and lower limb joints); 3. Muscle strength loss; 4. Gross Motor Function Classification grades. For example, patients with diagnosis of two or more developmental delays, including mental or cognitive, language, movement and socio-emotional, or have been obtain a medical report on comprehensive developmental delay could acquire disability card [24].

The exclusion criteria for this study were CP diagnosed when patients were older than 18 years old (n = 1519) or incomplete data(n = 108).

### Outcomes and predictor variables

The main outcome was the occurrence of severe pneumonia, defined by the inpatient pneumonia code (ICD-9-CM codes: 480–486 and 507.0–507.8) for more than 5 days. 5 days was chosen according to Zhang’s systematic analysis, the mean lengths of stay in hospital for children severe pneumonia is 5.8 days [25]. Patients’ sociodemographic characteristics, including CP diagnosis age (base on RCIPD), sex, residential area (Northern, Central, Southern and others), and hospital level (medical center, regional and others), were obtained from their initial enrollment data. Hospital level was categorized by the Ministry of Health and Welfare in Taiwan based on the staff teaching quantity and quality, physicians’ training capacity, the number of beds, the diversity of specialties, and the rate of emergency department visits. The qualification of the hospital-level will be evaluated periodically. Medical centers in Taiwan have the most physicians’ training implementation and medical care burden.

The comorbidities associated with severe pneumonia were retrieved by ICD-9-CM code from inpatient claims and ambulatory data. The ICD-9-CM codes were as follows: hearing loss (ICD-9-CM code: 389), asthma (ICD-9-CM code: 493), epilepsy (ICD-9-CM code: 345), diabetes mellitus (DM; ICD-9-CM code: 250), perinatal complications (ICD-9-CM codes: 760–764, 766–779, and V137), cerebral vascular accident (CVA; ICD-9-CM codes: 430–438), GERD (ICD-9CM codes: 530.85, 530.11, and 530.81), dysphagia (ICD-9CM code: 787.2), pressure ulcer (ICD-9CM code: 707.0), chronic liver disease (ICD-9CM code: 571), and intellectual disability (ICD-9CM codes: 317, 318, and 319). These comorbidities were included in the analysis if they occurred in an inpatient setting or in ≥3 ambulatory care claims for a chronic disease (or ≥1 ambulatory care claim for an acute disease).

### Statistical analysis

The categorical variables for the study groups were compared using the chi-square test, and continuous variables were analyzed using one-way analysis of variance. The multivariable logistic regression model was used to assess variables associated with severe pneumonia, considering the demographic characteristics and comorbidities.

In addition, we constructed the nomogram plot to estimate the severe pneumonia probability. A nomogram was plotted to determine the numerical probability of severe pneumonia based on significant variables (including characteristics and comorbidities) selected from the multivariate logistic regression model. On the basis of the estimated beta coefficients, we ranked the estimated the effects of each variable. Finally, calibration curves were plotted to assess the nomogram calibration, along with the Hosmer–Lemeshow (H–L) test. A significant test statistic implied that the model was not calibrated perfectly.

All statistical analyses were performed using Statistical Analysis Software (SAS; version 9.4; SAS System for Windows) and SPSS (version 20; SPSS Inc., Chicago, IL). A *p* value of <0.05 was considered statistically significant.

## Results

The eligible study participants were 6,356 CYP (59%:male) with newly diagnosed severe CP (median diagnosis age, 3.025 years), including 2,135 CYP (33.59%) with severe pneumonia (CP mean diagnosis age, 3.6 ± 3.9 years); 2,999 CYP (47.18%) without severe pneumonia (CP mean diagnosis age, 5.8 ± 4.6 years); and 1,222 CYP (19.23%) with severe pneumonia and hospitalization for 1–4 days (CP mean diagnosis age, 3.9 ± 3.6 years). The baseline characteristics of patients according to the subgroups of pneumonia are listed in Table 1. Among all relevant variables, CYP with severe pneumonia were significantly younger than those without severe pneumonia (3.6 ± 3.9 years vs. 5.8 ± 4.6 years; *p* < 0.001). In addition, comparing between the severe pneumonia and non-pneumonia groups revealed that CYP with CP along with severe pneumonia were more likely to be male (62% vs. 38%, *p* < 0.01) and have more comorbidities, except DM and intellectual disability.

**Table 1:** Baseline characteristics of children and young people with severe cerebral palsy according to subgroups of pneumonia, n = 6356.

Variables	None	1~4 days	≥ 5 days	P value
	N = 2999 (%)	N = 1222 (%)	N = 2135 (%)	
Age^a^ (Mean ± SD)	5.8±4.6	3.9±3.6	3.6±3.9	<0.001
Gender				0.001
Male	1691 (56%)	745 (61%)	1314 (62%)	
Female	1308 (44%)	477 (39%)	821 (38%)	
Hospital characteristics				<0.001
Medical center	1751 (58%)	705 (58%)	1371 (64%)	
Regional/Others	1248 (42%)	517 (42%)	764 (36%)	
Region				<0.001
North	1613 (54%)	568 (46%)	1005 (49%)	
Middle	612 (20%)	316 (26%)	516 (24%)	
South/others	774 (26%)	338 (28%)	564 (26%)	
Hearing loss				<0.001
Yes	276 (9%)	185 (15%)	315 (15%)	
No	2723 (91%)	1037 (85%)	1820 (85%)	
Epilepsy				<0.001
Yes	715 (24%)	540 (44%)	1086 (51%)	
No	2284 (76%)	682 (56%)	1049 (49%)	
Asthma				<0.001
Yes	120 (4%)	174 (14%)	281 (13%)	
No	2879 (96%)	1048 (86%)	1854 (87%)	
Perinatal complications				<0.001
Yes	919 (31%)	586 (48%)	1094 (51%)	
No	2080 (69%)	636 (52%)	1041 (49%)	
CVA				<0.001
Yes	266 (9%)	131 (11%)	269 (13%)	
No	2733 (91%)	1091 (89%)	1866 (87%)	
DM				0.910
Yes	13 (1%)	6 (1%)	11 (1%)	
No	2986 (99%)	1216 (99%)	2124 (99%)	
Intellectual disability				0.077
Yes	367 (12%)	177 (15%)	255 (12%)	
No	2632 (88%)	1045 (85%)	1880 (88%)	
GERD				<0.001
Yes	54 (2%)	39 (3%)	127 (6%)	
No	2945 (98%)	1183 (97%)	2009 (94%)	
Dysphagia				<0.001
Yes	30 (1%)	22 (2%)	64 (3%)	
No	2969 (99%)	1200 (98%)	2071 (97%)	
Pressure ulcer				0.006
Yes	9 (1%)	1 (0.1%)	16 (1%)	
No	2990 (99%)	1221 (99.9%)	2119 (99%)	
Chronic liver disease				0.025
Yes	14 (1%)	9 (1%)	24 (1%)	
No	2985 (99%)	1213 (99%)	2111 (99%)	

Abbreviations: SD = standard deviation; CVA = Cerebral Vascular Accident; DM = Diabetes Mellitus; GERD = Gastroesophageal Reflux Disease

^a^ Age: The age of newly diagnosed cerebral palsy

The characteristics of the patients and the results of univariate logistic regression analysis are presented in Table 2. We compared with and without severe pneumonia (inpatient care ≥ 5 days and none) among CYP with severe CP. The crude odds ratio (OR) for severe pneumonia in CP children diagnosis aged <3.1 years compared with those aged ≥3.1 years having CP was 2.84 (95% CI: 2.53–2.3.18, *p* < 0.001). Similarly, CYP with severe CP have comorbidities of hearing loss, DM, dysphagia, pressure ulcer, GERD, asthma, seizures, and perinatal complications were at an increased risk of developing severe pneumonia.

**Table 2:** Univariate logistic regression analysis for severe pneumonia among children and young people with severe cerebral palsy with ≥5 days inpatient care and none, n = 5134.

Variables	Beta	OR (95% CI)	P value
Age group (Mean = 3.1)			
0~3.1 year	1.042	2.84 (2.53–2.3.18)	<0.001
>3.1 year		1	
Gender			
Female		1	
Male	0.213	1.24 (1.11–1.37)	<0.001
Hearing loss			
Yes	0.535	1.71 (1.44–2.03)	<0.001
No		1	
Epilepsy			
Yes	1.196	3.31 (2.94–3.73)	<0.001
No		1	
Asthma			
Yes	1.291	3.64 (2.91–4.54)	<0.001
No		1	
Perinatal complications			
Yes	0.866	2.38 (2.12–2.67)	<0.001
No		1	
CVA			
Yes	0.393	1.48 (1.24–1.77)	<0.001
No		1	
DM			
Yes	0.174	1.19 (0.53–2.66)	0.673
No		1	
Intellectual disability			
Yes	0.028	1.03 (0.87–1.22)	0.751
No		1	
GERD			
Yes	1.230	3.42 (2.48–4.73)	<0.001
No		1	
Dysphagia			
Yes	1.118	3.06 (1.98–4.74)	<0.001
No		1	
Pressure ulcer			
Yes	0.920	2.51 (1.11–5.69)	0.028
No		1	
Chronic liver disease			
Yes	0.885	2.424 (1.25–4.69)	0.009
No		1	

Abbreviation: OR = Odd Ratio; CVA = Cerebral Vascular Accident; DM = Diabetes Mellitus; GERD = Gastroesophageal Reflux Disease.

The results of the stepwise logistic regression analysis are presented in Table 3. After adjustment for age, sex, and comorbidities, CP diagnosis age <3.1 years (OR: 2.49; 95% CI: 2.19–2.85; *p* < 0.001), male sex (OR: 1.22; 95% CI: 1.08–1.38; *p* = 0.002), and comorbidities of epilepsy (OR: 3.19; 95% CI: 2.81–3.63; *p* < 0.001), asthma (OR: 3.60; 95% CI: 2.85–4.58; *p* < 0.001), perinatal complications (OR: 1.60; 95% CI: 1.40–1.82; *p* < 0.001), GERD (OR: 1.56; 95% CI: 1.11–2.11; *p* = 0.012), and pressure ulcer (OR: 3.97; 95% CI: 1.66–9.45; *p* = 0.002) were identified as independent risk factors for severe pneumonia.

**Table 3:** Stepwise logistic regression analysis for severe pneumonia among children and young people with ≥ 5 days inpatient care and none, n = 5134.

Variables	Beta	OR (95% CI)	P value
Age group (Mean = 3.1)			
0~3.1 year	0.915	2.49 (2.19–2.85)	<0.001
>3.1 year		1	
Gender			
Female		1	
Male	0.196	1.22 (1.08–1.38)	0.002
Epilepsy			
Yes	1.161	3.19 (2.81–3.63)	<0.001
No		1	
Asthma			
Yes	1.282	3.60 (2.85–4.58)	<0.001
No		1	
Perinatal complications			
Yes	0.468	1.60 (1.40–1.82)	<0.001
No		1	
GERD			
Yes	0.447	1.56 (1.11–2.21)	0.012
No		1	
Pressure ulcer			
Yes	1.380	3.97 (1.66–9.45)	0.002
No		1	

### Prognostic nomogram development and calibration

**Fig 1:**
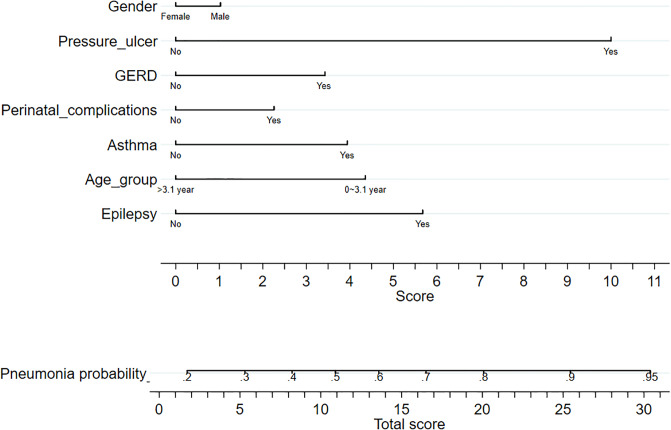
Nomogram plot for predicting severe pneumonia in CP children. For an individual patient, each variable corresponds to a point in the 8^th^ row (names “score”). The total points were summed up by all points and are indicated in the 10^th^ row (the bottom row). Drawing a vertical line from total point to the 9^th^ row will show the corresponding probability of pneumonia.

Fig 1 present prognostic nomograms with independent risk factors for severe pneumonia in children with severe CP. Each significant variable listed in Table 3 was assigned a score on the point scale. A straight line could be drawn to estimate the probability of severe pneumonia at each time point by summing up the total score and locating it on the total point scale.

For example, if the patient was male and with GERD and pressure ulcer, we located a patient’s gender on the relevant axis first. Next, we draw a straight line downward to the point axis (8^th^ row, named “Score”) to obtain the points based on gender (male was 1 point). Then we repeated this course for age variable (CP diagnosis age is 2 years old:4.5 points), GERD variable (3.5 points) and pressure ulcer variable (10 points). After that, we summed up all the points (19 points) to obtain the “Total score” (the bottom row). Finally, we draw a straight line upward from the 10^th^ row to obtain the probability of developing pneumonia in the 9^th^ row. That is, a male CP who diagnosis age is 2-year-old patient with pressure ulcer and GERD history has a nearly 80% probability of serve pneumonia.

**Fig 2:**
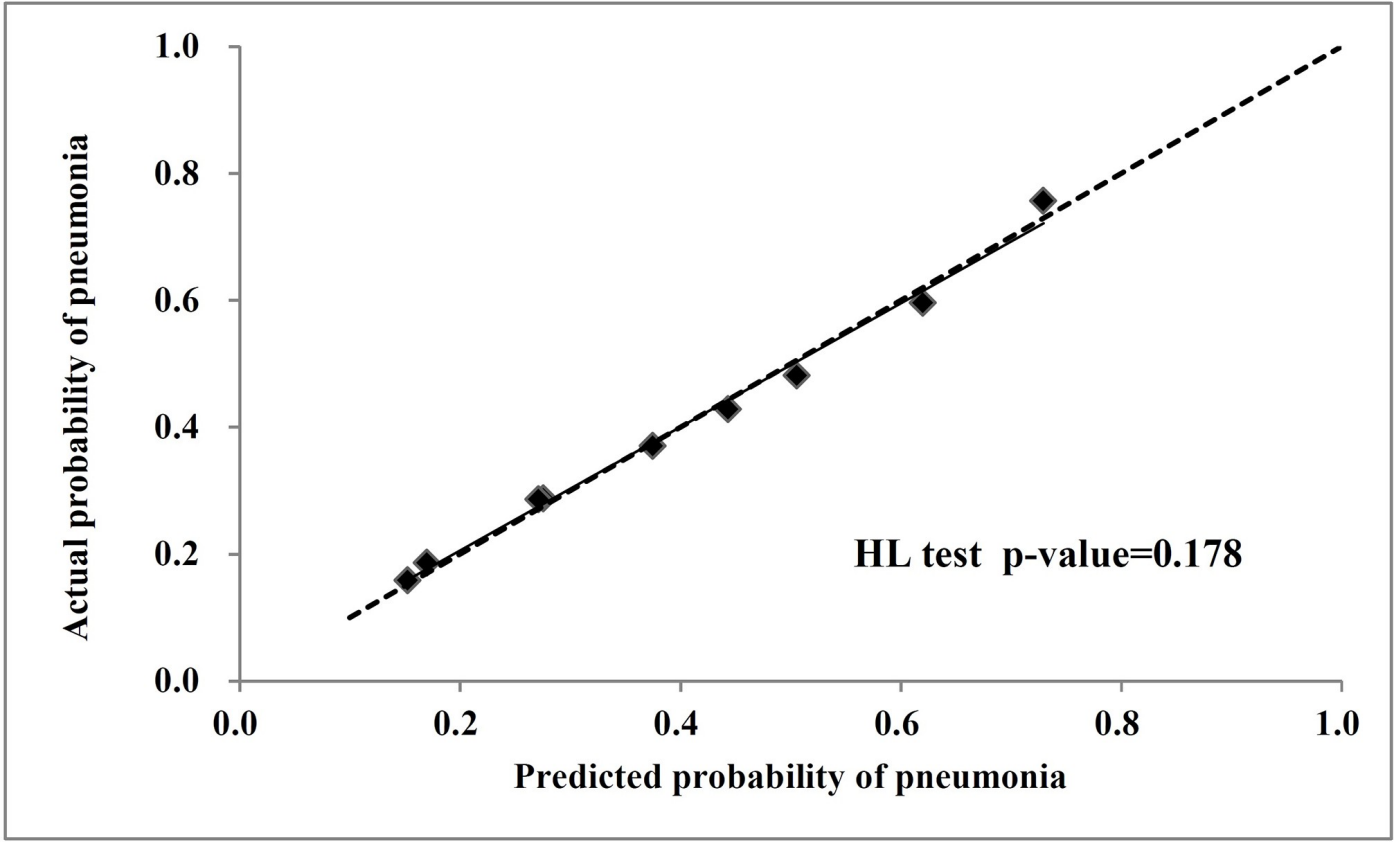
The calibration curve for the radiomics nomogram plot. Dashed line indicates ideal reference line where predicted probabilities would match the observed proportions (the diagonal square) and, of which a closer fit to the diagonal dotted line represents a better prediction. The Hosmer-Lemeshow test showed the p-value is 0.178.

Fig 2 showed a calibration curve that the predicted probabilities of pneumonia (the diagonal square) matched the reference line. The dashed line is an ideal reference line representing the predicted probabilities of pneumonia would match the observed proportions. Additionally, a closer fit to the dashed line represents a better prediction. The H-L test p-value was 0.1783 (p > 0.05), which demonstrated good agreement between prediction and observation. It also indicates the performance of our model is good.

## Discussion

To the best of our knowledge, this is the first cohort study to predict severe pneumonia risk in children with severe CP through nomograms using nationwide population-based data. We classified the risk based on the medical comorbidities and sociodemographic factors. The major finding of this study was that CP diagnosis age < 3 years; male sex; and having comorbidities namely epilepsy, asthma, perinatal complications, GERD, and pressure ulcer were significantly correlated with severe pneumonia in children with severe CP. Second, the nomogram was found to be a suitable and useful tool for predicting the probability of severe pneumonia.

This study has several strengths. This nationwide population-based cohort study included CYP of all ages with newly diagnosed severe CP (<18 years old) between 1997 and 2013, had a longer follow-up period, and the first study to use a prognostic nomogram for CYP CP severe pneumonia prediction.

In past microbiological studies, the most relevant bacterial stains causing children pneumonia included *Streptococcus pneumoniae* and *Haemophilus influenzae* type B, followed by *Staphylococcus aureus* and *Mycoplasma pneumoniae* [16]. Bacterial pathogens associated with WHO-defined very severe pneumonia (the most advanced form of the disease) were: *Streptococcus pneumoniae* and *Staphylococcus aureus*, followed less commonly by *Haemophilus influenzae*, *Escherichia coli*, and *Pseudomonas aeruginosa* [16].

Pressure ulcer was found to be the leading factor for severe pneumonia occurrence. This may be because they share many pathogenic factors and may interact with each other. pressure ulcer has been known to be caused by immobility, sensory loss, and malnutrition [26] and occurred more frequently among children with a deteriorated neurological condition such as CP [27]. According Leonard’s research, malnutrition and higher Gross Motor Function Classification System (GMFCS) level were risk factors for pneumonia [28]. Despite that nutritional status and GMFCS were not accessible in Taiwan NHIRD, diagnosis of pressure ulcer could be an indicator of poorer functional level. Therefore, pressure ulcer is the most dominant factor for severe pneumonia occurrence. The prevalence of pressure ulcer in the current study was low. We presume the reason is that abnormal neuromuscular development among YCP with CP hinders independent movement and withdrawal from pressure [29].

Asthma was found to be the dominant factor for pneumonia in the present study. Some studies have revealed that children with asthma had sustained increase in risk for invasive *Pneumococcal pneumonia* infections [30–32]. The increased risk was attributed to the structural changes arising around the trachea, bronchi, and bronchioles, leading to chronic inflammation [33]. Another possible reason may due to higher *Streptococcus pneumoniae* nasopharyngeal carriage and primary immunodeficiencies in children with asthma.[34] Additionally, inhaled corticosteroids were associated with oropharyngeal *S*. *pneumoniae* colonization in children with asthma [35].

Younger CP diagnosis age in Children were significantly more risk for getting severe pneumonia, which is consistent with the result of a previous retrospective study.[36] The younger a person is when CP is diagnosed, the more severe the CP is, and there will be more complications, including pneumonia. One study investigating invasive pneumococcal infections in infants and young children in Santiago also found a higher prevalence of pneumonia in younger children (<6 months) [37].

GERD has been considered a factor associated with the long-term risk of pneumonia [38]. Occult micro aspiration has been reported as the key pathological factor connecting GERD and lung disease [39]. Furthermore, spasticity of abdominal muscles causing increased intra-abdominal pressure in patients with severe CP was noted to contribute to pneumonia [8].

Pneumonia has been widely recognized as a complication of seizures. A population-based study on children admitted in intensive care revealed a similar finding that epilepsy was a significant risk factor for pneumonia [40]. The major cause was the aspiration of secretions, when seizure hindered the airway protective reflexes. Second, aspiration occurred frequently in supine position during postictal recovery and increased orotracheal secretions in the postictal state [41].

Perinatal complications were associated with an increased risk of severe pneumonia among CYP CP. The association between perinatal complications and CP have been established [42] and result in younger CP diagnosis. Early diagnosis indicates greater severity of CP with poorer outcomes. As mentioned above, younger CP diagnosis age in Children were significantly more risk for getting severe pneumonia. Moreover, Perinatal complications, such as chorioamnionitis or fetal asphyxia leading to amniotic fluid bacterial infection or colonization of the birth canal were associated with pneumonia [43]. Perinatal anoxia or traumatic brain injury have been demonstrated a linkage to swallowing dysfunction [44]. Without intact swallowing function as integrated epiglottic and cough reflexes, people cannot avoid aspiration and expel infected secretions. Therefore, underlying perinatal complications and CP both predispose children to pneumonia.

Dysphagia has been considered a common symptom in children with CP and tend to present food aspiration, malnutrition, and respiratory infections [45]. Blackmore et al suggested that the strongest modifiable risk factor for respiratory-related hospitalizations in pediatric CP was oropharyngeal dysphagia [10]. However, our study revealed a contradictory result. This may be attributed to the incomparability of the study definition that we used (ICD-9) for diagnosis with low prevalence of dysphagia. Because video-fluoroscopic swallowing study and fiber-optic endoscopic examination were time-efforts consuming and high patient’s cooperation demand in Taiwan. Thus, few patients received this standard evaluation.

Comparing between the severe pneumonia and non-pneumonia groups revealed that CYP with CP along with severe pneumonia were more likely to be male (62% vs. 38%, p < 0.01). According to Nathan’s children prospective cohort study, bacterial pneumonia was seen more in males [46]. We think some factors play the role in it. First, male children usually have poor hygiene and sanitary habits, and that both habits are risk factors for pneumonia. Additionally, male children are more likely to be exposed to outdoor air pollution due to more external activities than female children [47]. Second, the anatomical disparity in the respiratory tract may partially explain the different incidence of pneumonia between men and women. For example, peripheral airways are disproportionately narrowed in male’s early childhood, which can lead to lower respiratory tract infections [48]. Third, human lung development and pulmonary infection susceptibility were affected by altered estrogen and testosterone levels. Evidence have suggested an active role of estrogen in sexual dimorphism by presenting different estrogens levels in lung maturation, preservation, regeneration, alveoli development and surfactant synthesis. Female pulmonary surfactant production was manifested earlier than male [49]. Fourth, estrogen and androgen play the opposite way on immune responses after infection. Androgens in males cause extended susceptibility to infections. Inversely, estrogen makes females less vulnerable to some infectious [50]. Though sex hormone effect is not apparent in young children, it may partially illustrate the sex disparity on infections.

The nomogram was composed of several simple demographic and clinical factors, which may be useful for identifying patients with a high probability of developing severe pneumonia. The nomogram could be used to closely monitor CYP with severe CP and facilitate physicians or healthcare professionals in clinical care. Moreover, the nomogram may provide appropriate information and suggestions to the family of CYP with severe CP, facilitating early alert and transfer for medical management. Considering the public health viewpoint, policymakers are encouraged to enforce severe pneumonia risk screening in CYP with CP and to provide more integrated care such as a combination of medical care and rehabilitation therapy.

Several limitations for interpreting this study results existed. First, the Gross Motor Function Classification System (GMFCS) level was not accessible in Taiwan NHIRD, which is an indicator of poor functional level. GMFCS level V children do worse than those with lower levels of severity. CPY with CP classified at level V GMFCS had higher risk of hospital admissions than other GMFCS levels [10]. However, we used ICF, catastrophic illness certification card and disability cards to make our diagnosis more precise. Second, we could not obtain the birth body weight, nutrition status, parental educational level, breast feeding duration, and household environment, such as presence of cigarette smoke. Third, the prevalence of dysphagia, GERD and pressure ulcer were low result from that we used (ICD-9-CM codes) for diagnosis, and we should carefully interpret the result due to potential dropout bias and underestimation in our database. Presence of enteral feeding (i.e. nasogastric tube, nasoduodenal tube, gastrostomy tube, jejunostomy tube, gastrostomy/jejunostomy tube) were associated with much worse CP outcomes. But in the present study, we did not get the data. Fourth, because we only assessed severe CYP patients and we cannot apply the results to all CP population. Lastly, defining the pathogen of pneumonia can be difficult due to the challenge in collecting samples from young children’s lower respiratory tract and waived from contaminated during the procedure. Therefore, the diagnosis of pneumonia was defined by ICD-9 code and we cannot obtain the pathogens or causes of pneumonia in this database.

## Conclusion

Younger age, male sex, and history of pressure ulcer, GERD, asthma, seizures, and perinatal complications were found to be potential risk factors for severe pneumonia in CYP with severe CP, which can be influenced by different interventions in the future. The nomogram was found to be a useful tool for identifying CYP with severe CP having a high risk for severe pneumonia.

## Abbreviations

CVA: Cerebral vascular accident
DM: Diabetes mellitus
GMFCS: Gross Motor Function Classification System
ICD-9-CM: International Classification of Disease, Ninth Revision, Clinical Modification
ICF: International Classification of Functioning
NHI: National Health Insurance
NHIRD: National Health Insurance Research database
OR: Odds ratio
RCIPD: Registry for catastrophic illnesses patient database
